# A unique expression pattern of *LAG3* distinct from that of other immune checkpoints in diffuse large B‐cell lymphoma

**DOI:** 10.1002/cam4.6268

**Published:** 2023-06-16

**Authors:** Hyunjee Lee, Sang Eun Yoon, Seok Jin Kim, Won Seog Kim, Junhun Cho

**Affiliations:** ^1^ Department of Pathology Samsung Medical Center, Sungkyunkwan University School of Medicine Seoul South Korea; ^2^ Division of Hematology and Oncology, Department of Medicine Samsung Medical Center, Sungkyunkwan University School of Medicine Seoul South Korea

**Keywords:** CD274, diffuse large B‐cell lymphoma, immune checkpoint, LAG3, LAG‐3, PD‐L1

## Abstract

**Background:**

Although some patients with diffuse large B‐cell lymphoma (DLBCL) show a response to immunotherapy, there are still many who do not respond. This suggests that various immune checkpoints are complicatedly intertwined in the composition of the tumor microenvironment of DLBCL.

**Patients and Methods:**

To comprehensively understand the expression of various immune checkpoint genes in DLBCL, we performed NanoString assay in 98 patients to investigate 579 genes. In addition, we performed immunohistochemistry for LAG‐3 and PD‐L1 to compare the results with expression in NanoString assay.

**Results:**

As a result of hierarchical clustering of NanoString assay, 98 DLBCLs were classified into three tumor immune microenvironment clusters. Most immune checkpoint genes showed the highest expression in cluster A and the lowest in cluster C. However, the expression of *LAG3* was the highest in cluster C and the lowest in cluster A, showing an expression pattern opposite to that of other immune checkpoint genes. In Cluster A, the expression of genes related to T‐cell activity such as *CD8A* and *GZMB* was increased. In Cluster C, the expression of genes related to major histocompatibility complex molecules was the highest. Immunohistochemical stains showed modest agreement with the NanoString results but did not help clustering.

**Conclusion:**

Our results show that the unique expression pattern of *LAG3* in DLBCL contrasts with that of other immune checkpoints. We suggest that the combination of anti‐PD‐1/PD‐L1 and anti‐LAG‐3 blockades in the immunotherapy of DLBCL patients can have a synergistic effect, improving the immunotherapy efficacy and outcome in DLBCL patients.

## 1 INTRODUCTION

The development and application of immunotherapy have improved the prognosis of patients with numerous solid tumors.^1^, ^2^, ^3^ The most widely used immunotherapies target the programmed death‐1 (PD‐1)/programmed death‐ligand 1 (PD‐L1) axis, associated with the T‐cell activity. Treatments targeting various immune checkpoints, such as cytotoxic T‐lymphocyte antigen‐4 (CTLA‐4) and lymphocyte‐activation gene 3 (LAG‐3), are also used or are in clinical trials.^4^, ^5^, ^6^, ^7^

Despite continuous efforts to use it to treat lymphomas, immunotherapy has only been effective for classic Hodgkin lymphoma (cHL)^8^, ^9^, ^10^, ^11^ and primary mediastinal large B‐cell lymphoma (PMLBL).^12^, ^13^ Even after treating diffuse large B‐cell lymphoma (DLBCL)—the most common non‐Hodgkin lymphoma—with rituximab, cyclophosphamide, doxorubicin, vincristine, and prednisone (R‐CHOP) therapy, the 5‐year survival rate is approximately 70%.^14^ Therefore, we need to improve the prognosis of DLBCL patients through immunotherapy. Immunotherapy is effective in a subset of DLBCL patients; however, the efficacy of anti‐PD‐1/PD‐L1 blockade remains unproven in a large number of patients, even in tumors with PD‐L1 expression.^15^, ^16^ Resistance to anti‐PD‐1/PD‐L1 therapy poses a challenge in the treatment of DLBCL. The mechanism of resistance to anti‐PD‐1/PD‐L1 therapy is unclear but might be because the tumor immune microenvironment (TIME) in DLBCL is not simple, and the interaction of very complex factors is intertwined. Therefore, combination immunotherapy targeting two or more immune checkpoints has been studied in many solid tumors.^17^, ^18^

Chimeric antigen receptor T (CAR‐T) cell therapy has been used to treat lymphoma. CAR‐T cell therapy allows us to control the T‐cells attacking tumor cells via expressing specific receptors through immunoediting.^19^ Knocking out the receptor that receives the signal from the inhibitory immune checkpoint in CAR‐T cells enables CAR‐T cells to maintain a constant antitumor effect without TIME interference. To increase the efficacy of CAR‐T cell therapy, it is necessary to precisely understand the inhibitory signal present in the TIME of DLBCL.

To understand the TIME landscape, it is essential to examine the expression patterns of various immune checkpoints and inflammatory activity markers. Therefore, in this study, we attempted to comprehensively identify the activity of inflammation and various immune checkpoint genes in DLBCL using the NanoString assay consisting of a panel of genes related to human immunity. We found that the expression of the *LAG3* gene is increased in a pattern opposite to that of most other immune checkpoint genes and is associated with immune depletion.

## 2 PATIENTS AND METHODS

### 2.1 Patient selection

Patients diagnosed with DLBCL at Samsung Medical Center, Seoul, Korea, between January 2011 and December 2015 were enrolled in the study. From these, we selected 98 cases from various organs to compare TIME, and for whom formalin‐fixed paraffin‐embedded (FFPE) blocks were available. Based on the WHO classification^14^ and International consensus classification,^20^ DLBCL, not otherwise specified (NOS), primary diffuse large B‐cell lymphoma of the central nervous system (PCNSL), primary large B‐cell lymphoma of the testis (PTL), EBV‐positive DLBCL (EBV DLBCL), PMLBL, and intravascular large B‐cell lymphoma (IVLBL) were found in 62 (63.3%), 17 (17.3%), 6 (6.1%), 6 (6.1%), 4 (4.1%), and 3 cases (3.1%), respectively. There was no post‐transplant lymphoproliferative disorder case. Clinicopathological information, including age, sex, location, Ann Arbor stage, and survival data was evaluated by reviewing electronic medical records. All methods were carried out following the Helsinki Declaration, and the Institutional Review Board of Samsung Medical Center approved all protocols of this study (IRB file number: 2021‐01‐093‐004). A waiver of written informed consent was granted by the Institutional Review Board.

### 2.2 NanoString nCounter assay

We analyzed RNA expression using nCounter® technology from NanoString in all 98 cases. The nCounter® Human Immunology V2 Panel with 579 human immune signature genes and 15 housekeeping genes (NanoString Technologies) was used for the NanoString nCounter assay. Total RNA was extracted from 3 to 4 μm thick FFPE tissue sections from representative blocks using the High Pure RNA Paraffin kit (Roche Diagnostics). RNAs (200 ng) were hybridized to the target sequence‐specific capture probes and fluorescence‐labeled reporter probes. The mRNA‐probe complexes were washed, immobilized, and quantified using fluorescence imaging. Two‐step normalization was performed for the gene expression matrix to remove the batch effect of nCounter gene expression. First, we performed within‐normalization using the NanoStringNorm R package with options CodeCount+Sum, Background=mean, and SampleContent=total.sum and adjusted the outliers to the median value with the outlier R package.^21^ Next, gene expression matrices spanning two batches were rescaled by between‐normalization using the edgeR R package, and log_10_ transformed expression was considered the final gene expression matrix.^22^ We removed 104 genes with very low mean expression (<1, < normalized data 10) from the analysis, created a heatmap for 475 genes and performed hierarchical clustering (https://software.broadinstitute.org/morpheus/).

### 2.3 Immunohistochemistry

Based on the results of the NanoString assay, we performed immunohistochemistry (IHC) for LAG‐3 (clone EPR4392; Abcam) and PD‐L1 (clone SP142; Ventana). All slides were scanned using a Pannoramic 1000 digital slide scanner (3DHistech). An INFINITT DPS (INFINITT Healthcare) was used as the image viewing system. Images with an area of 0.37 mm^2^ were captured for two representative areas to measure the degree of infiltration of LAG‐3‐positive cells in tumors. The number of positive cells and the ratio (%) of positive cells to total cells in the captured images were investigated using QuPath software.^23^ A combined positive score (CPS) (the number of positive tumor cells, lymphocytes and macrophages divided by the total number of viable tumor cells, multiplied by 100) system was used to evaluate PD‐L1 expression. One pathology resident (HL) and one hematopathologist (JC) independently measured the CPS of all PD‐L1 stains, and the scores of cases with discrepancies were established by consensus.

### 2.4 Statistical analysis

The SPSS 27.0 statistical software program (IBM Corporation) was used for the statistical analyses. Pearson's chi‐square test was used for crossover analysis of clinicopathological features. A Kaplan–Meier curve (log‐rank test) was used for survival analysis. Pearson's correlation test was used to analyze the correlation between two continuous variables. Kruskal‐Wallis test was performed to compare two or more groups of continuous variables. *p* < 0.05 was considered statistically significant.

## 3 RESULTS

### 3.1 Patients' characteristics

The male‐to‐female ratio in the 98 patients was 63:35 (1.8:1), and the median age was 58 years (range, 3–87). Ann Arbor stages I–II and III–IV were present in 67 (68.4%) and 31 (31.6%) patients, respectively. In DLBCL NOS, germinal center B‐cell (GCB) type were 26 (44.1%) and non‐GCB type were 33 (55.9%) cases. Based on the primary organ of tumor, 18, 24, and 4 patients had tumors in the lymph nodes, immune‐privileged organs (brain and testis), and mediastinum, respectively, and the remaining 52 had tumors in other extranodal sites.

### 3.2 NanoString nCounter assay: 1. hierarchical clustering

**FIGURE 1:**
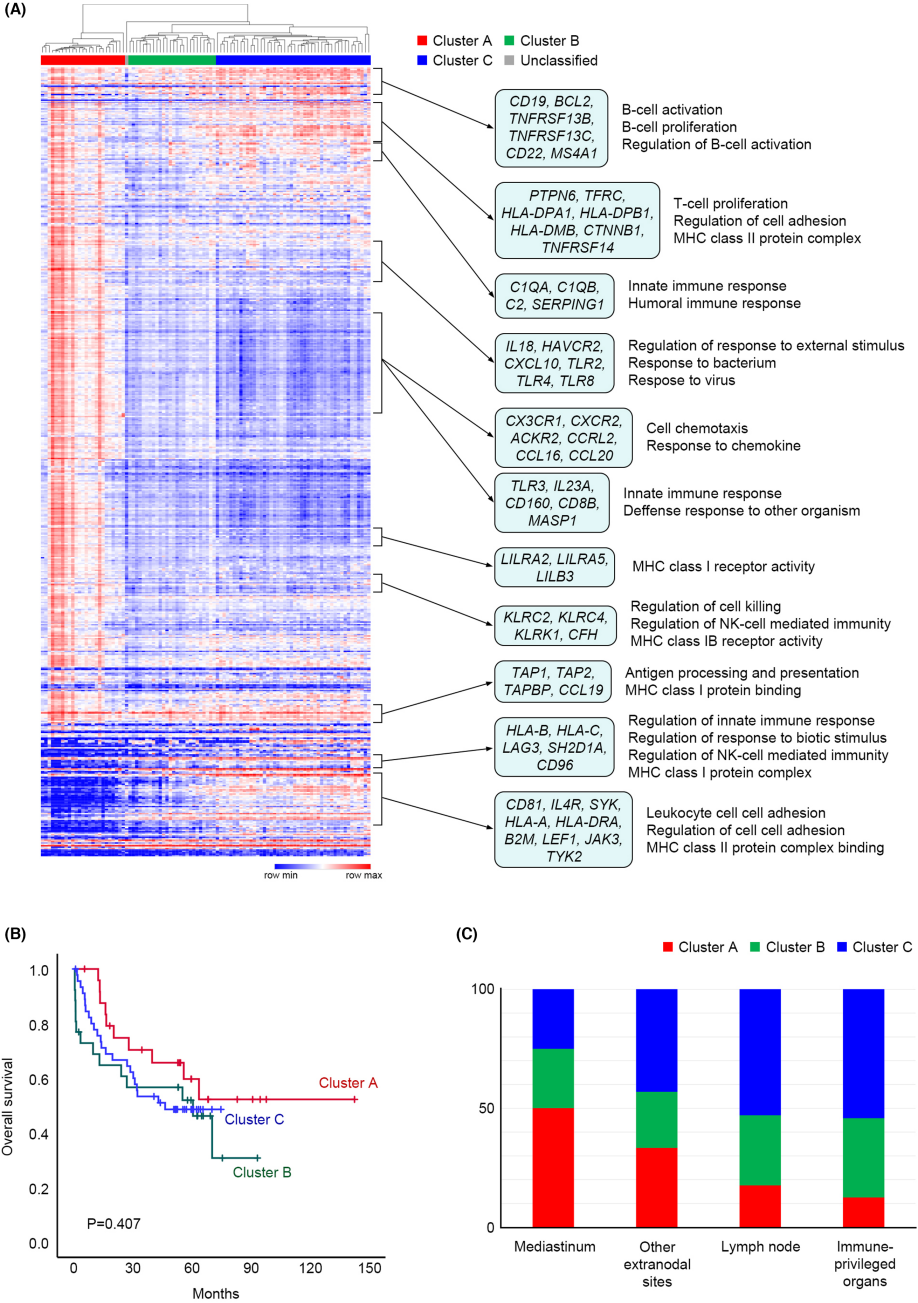
(A) A heatmap and hierarchical clustering of NanoString assay results. NanoString clusters A, B, and C included 25, 26, and 45 cases, respectively. Some representative genes and gene set enrichment analysis results are shown on the right side of the heatmap. (B) Kaplan–Meier curves of overall survival for each cluster. No significant prognostic differences were observed between the three clusters. (C) Bar graphs comparing the composition of each cluster by tumor location. Although not statistically significant, cluster A was found more frequently in lymphomas in mediastinum, and cluster C in lymphomas in immune‐privileged organs.

As a result of unsupervised hierarchical clustering, 98 cases were classified into three clusters (A, B, and C). Clusters A, B, and C had 25, 26, and 45 patients, respectively; the remaining 2 cases were unclassifiable. (Figure 1A) In the Kaplan–Meier curve, clusters A, B, and C showed no significant differences in prognosis. (Figure 1B) The three clusters showed no significant differences in sex, age, diagnosis, cell of origin, or Ann Arbor stage. (Table 1) DLBCL from the brain and testis, the immune‐privileged organs, had a high rate of cluster C and a low rate of cluster A, and tumors from the mediastinum and spleen showed a high rate of cluster A but no statistical significance between the tumor organs and NanoString clusters. (Figure 1C) Even when hierarchical clustering was performed on only 60 DLBCL NOS cases, they were also classified into three clusters, which showed a very high concordance with the clustering results of all cases (Table 2).

**TABLE 1:** Clinicopathological characteristics of diffuse large B‐cell lymphoma patients according to NanoString clusters (*N* = 96).

	Number (%)	NanoString cluster			*p* value
		A	B	C	
		*N* (%)	*N* (%)	*N* (%)	
Sex					
Male	62 (64.6)	15 (60.0)	16 (61.5)	31 (68.9)	0.705
Female	34 (35.4)	10 (40.0)	10 (38.5)	14 (31.1)	
Age					
<60	52 (54.2)	15 (60.0)	15 (57.7)	22 (48.9)	0.613
≥60	44 (45.8)	10 (40.0)	11 (42.3)	23 (51.1)	
Diagnosis					
DLBCL NOS	60 (62.5)	18 (30.0)	14 (23.3)	28 (46.7)	0.701
PCNSL	17 (17.7)	2 (11.8)	5 (29.4)	10 (58.8)	
PTL	6 (6.3)	0 (0.0)	3 (50.0)	3 (50.0)	
PMLBL	4 (4.2)	2 (50.0)	1 (25.0)	1 (25.0)	
EBV DLBCL	6 (6.3)	2 (33.3)	2 (33.3)	2 (33.3)	
IVLBL	3 (3.1)	1 (33.3)	1 (33.3)	1 (33.3)	
Cell of origin (in NOS)					
GCB	26 (45.6)	9 (34.6)	6 (23.1)	11 (42.3)	0.901
non‐GCB	31 (54.4)	9 (29.0)	8 (25.8)	14 (45.2)	
Ann Arbor stage					
I–II	66 (68.8)	19 (76.0)	16 (61.5)	31 (68.9)	0.538
III–IV	30 (31.3)	6 (24.0)	10 (38.5)	14 (31.1)	
Organ					
Lymph node	17 (17.7)	3 (17.6)	5 (29.4)	9 (52.9)	0.462
Other extranodal sites	51 (53.1)	17 (33.3)	12 (23.5)	22 (43.1)	
Immune‐privileged organs	24 (25.0)	3 (12.5)	8 (33.3)	13 (54.2)	
Mediastinum	4 (4.2)	2 (50.0)	1 (25.0)	1 (25.0)	

Abbreviations: DLBCL NOS, diffuse large B‐cell lymphoma, not otherwise specified; EBV DLBCL, EBV‐positive diffuse large B‐cell lymphoma; IVLBL, intravascular large B‐cell lymphoma; GCB, germinal center B‐cell type; PCNSL, primary diffuse large B‐cell lymphoma of the central nervous system; PMLBL, primary mediastinal large B‐cell lymphoma; PTL, primary diffuse large B‐cell lymphoma of the testis.

**TABLE 2:** Comparison of clustering results for all cases (*n* = 98) and diffuse large B‐cell lymphoma, not otherwise specified cases (*n* = 60) only.

	DLBCL NOS clustering			Total
	Cluster A (%)	Cluster B (%)	Cluster C (%)	
All DLBCL clustering				
Cluster A (%)	18 (100)	0 (0.0)	0 (0.0)	18
Cluster B (%)	0 (0.0)	12 (85.7)	2 (14.3)	14
Cluster C (%)	0 (0.0)	0 (0.0)	28 (100)	28
Total	18	12	30	60

Abbreviations: DLBCL, diffuse large B‐cell lymphoma; NOS, not otherwise specified.

Gene expression profile analysis (Figure 1A) revealed that genes that showed high expression levels in all three clusters were mainly associated with B‐cell activation and B‐cell proliferation (*CD19, BCL2, TNFRSF13B, TNFRSF13C, CD22, MS4A1*, etc.), antigen processing and presentation, and major histocompatibility complex (MHC) class I protein binding (*TAP1, TAP2, TAPBP, CCL19*, etc.). Genes related to T‐cell proliferation (*PTPN6, TFRC, HLA‐DPA1, HLA‐DPB1, HLA‐DMB, CTNNB1, TNFRSF14*, etc.) or innate immune response (*C1QA, C1QB, C2, SERPING1*, etc.) were highly expressed in clusters A and C but showed low expression in cluster B. Numerous immune‐related genes were highly expressed in cluster A and showed low expression in clusters B and C. Some genes were highly expressed in cluster C and showed low expression in cluster A, and these genes were associated with the regulation of innate immune response (*HLA‐B, HLA‐C, LAG3, SH2D1A, CD96*, etc.), regulation of cell adhesion, and MHC class II protein complex binding (*CD81, IL4R, SYK, HLA‐A, HLA‐DRA, B2M, LEF1, JAK3, TYK2*, etc.).

### 3.3 NanoString nCounter assay: 2. immune checkpoint analysis

**FIGURE 2:**
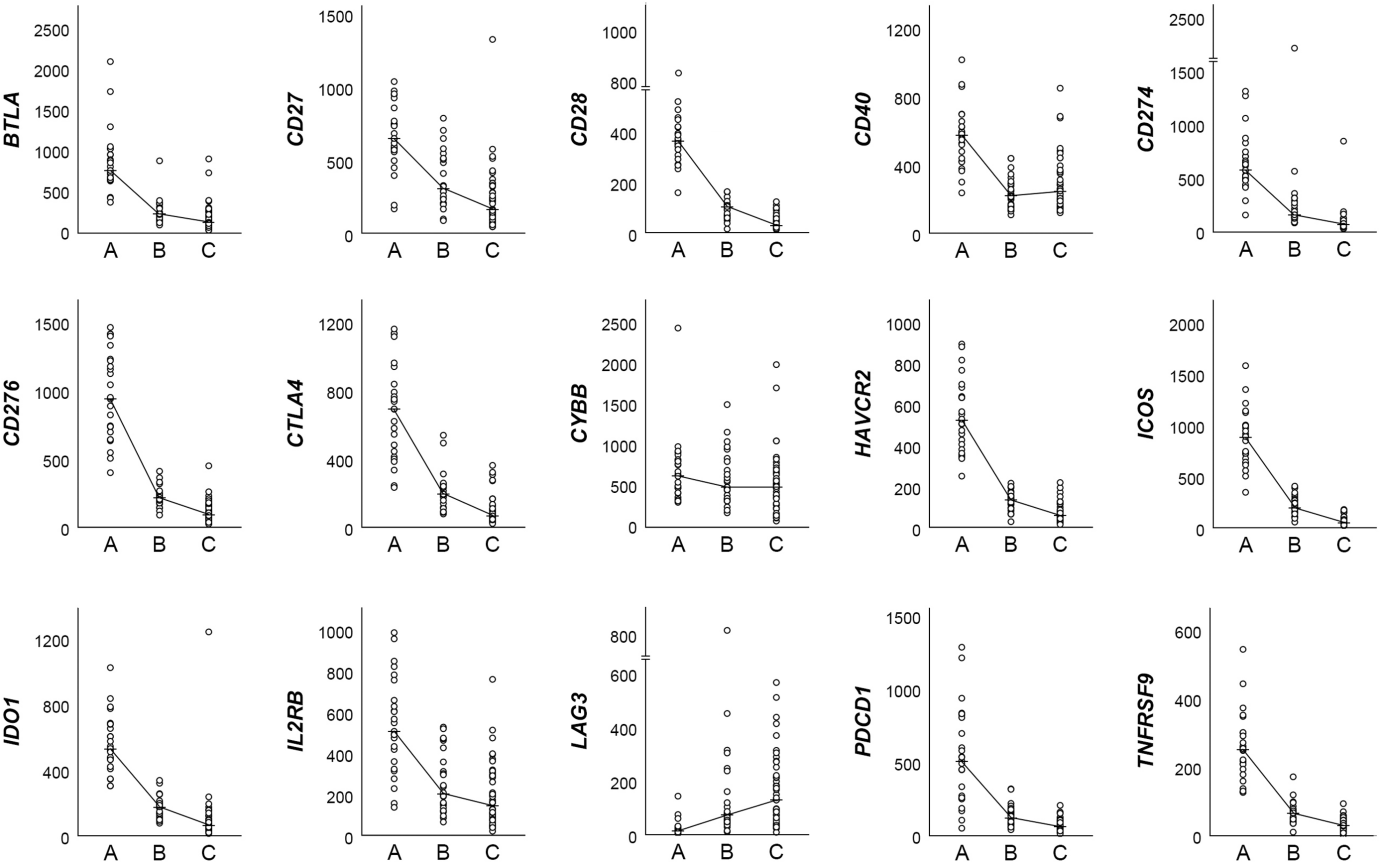
Comparison of the expression levels of 15 representative immune checkpoint genes across the clusters. The line graph represents the median value for each cluster. LAG3 (middle of the bottom low) shows a unique expression pattern distinct from that of other immune checkpoint genes.

To investigate the difference in expression of immune checkpoint genes according to the three clusters, 15 representative immune checkpoint genes (*BTLA, CD27, CD28, CD40, CD274, CD276, CTLA4, CYBB, HAVCR2, ICOS, IDO1, IL2RB, LAG3, PDCD1*, and *TNFRSF9*) were selected from those included in the NanoString gene panel to compare the degree of expression by clusters. (Figure 2) Among the 15 immune checkpoint genes, 14 genes showed the highest expression in cluster A in common, and among them, 12 genes, except *CD40* and *CYBB*, showed the lowest expression in cluster C. In contrast, only *LAG3* showed the lowest expression in cluster A and the highest expression in cluster C.

**FIGURE 3:**
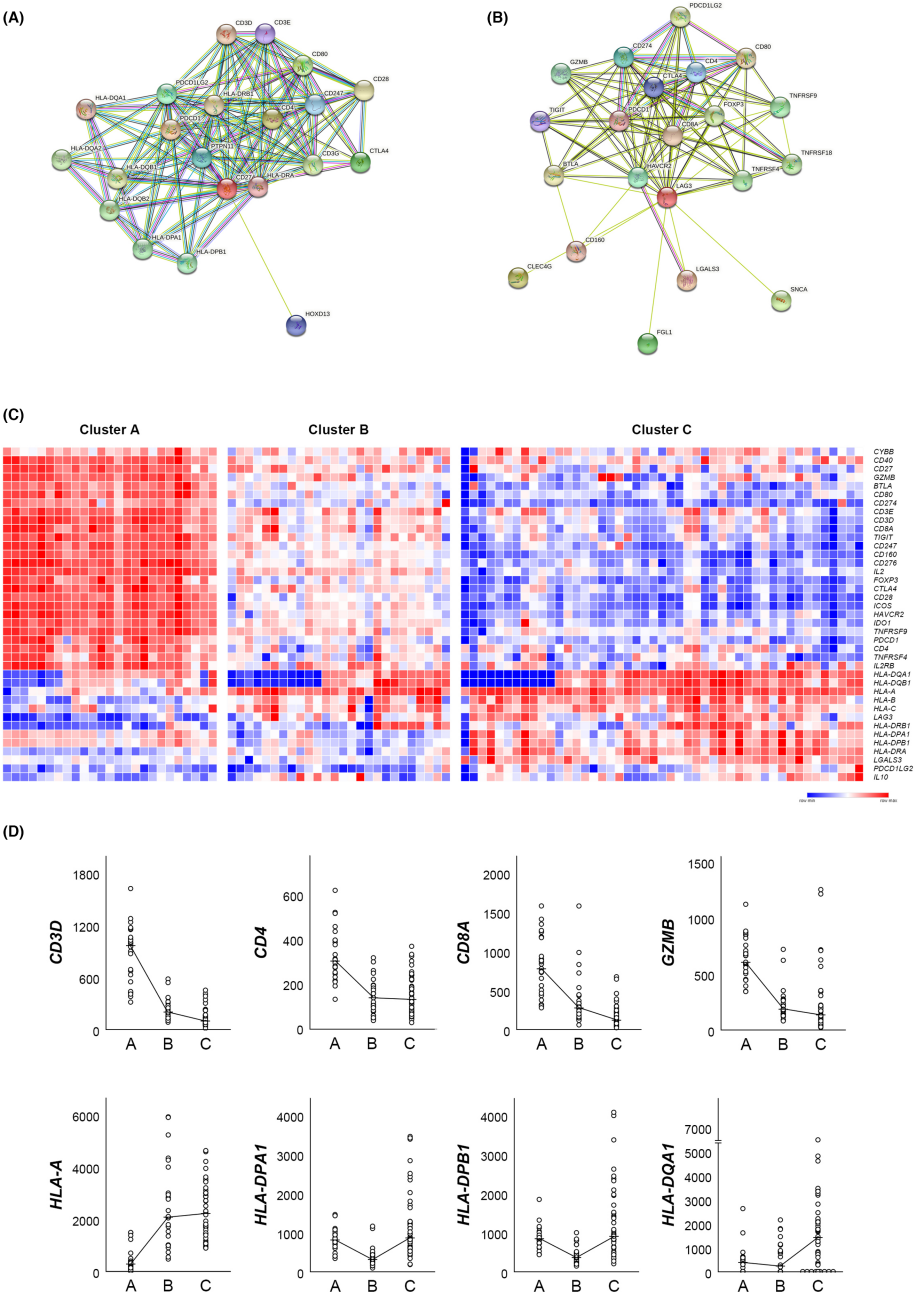
(A, B) STRING interaction analysis of the genes functionally related to *CD274* (A) and *LAG3* (B) (https://string‐db.org/). In both genes, the 20 genes with the highest functional relationship were shown in figures. (C) A reconstructed heatmap of Figure 1A only for the genes shown in A and B. (D) Comparison of the expression levels of four representative T‐cell activity‐associated genes and four major histocompatibility complex (MHC)‐associated genes across the clusters. T‐cell activity is the highest in cluster A, while MHC‐related gene expressions are the highest in cluster C.

Each of the 20 genes functionally related to *CD274*, selected by the authors to represent immune checkpoint genes with the highest expression in Cluster A, and *LAG3*, with the highest expression in Cluster C, were searched using the STRING website (https://string‐db.org/). *PDCD1, CD4, CD80, CTLA4, PDCD1LG2, PTPN11, CD247, HOXD13, CD3E, HLA‐DRA, HLA‐DQA1, CD3D, HLA‐DRB1, CD28, HLA‐DQB1, CD3G, HLA‐DQA2, HLA‐DQB2, HLA‐DPA1, HLA‐DPB1*, and *HLA‐DRB5* genes were searched as predicted functional partners of *CD274* (confidence >0.9, full STRING network). (Figure 3A) *LGALS3, CLEC4G, SNCA, FGL1, HAVCR2, CD274, CD4, CTLA4, TIGIT, PDCD1, CD160, CD8A, CD80, BTLA, PDCD1LG2, FOXP3, TNFRSF18, TNFRSF4, TNFRSF9, GZMB, LGALS9, IDO1, IL2*, and *IL10* genes were searched as predicted functional partners of *LAG3* (confidence >0.7, full STRING network). (Figure 3B) In Figure 3C, the heatmap in Figure 1A was reconstructed only for above 40 genes (15 representative immune checkpoint genes plus genes functionally related to *CD274* and/or *LAG3*). A number of immune checkpoint genes, including *CD274*, showed the highest expression in cluster A and the lowest expression in cluster C. By contrast, *HLA‐A, HLA‐B, HLA‐C, HLA‐DRB1, HLA‐DPA1, HLA‐DPB1*, and *HLA‐DRA* associated with MHC class I and II molecules showed the highest expression in cluster C along with *LAG3*. Representative T‐lymphocyte‐related genes (*CD3D, CD4, CD8A*, and *GZMB*) showed the highest expression in cluster A and the lowest expression in cluster C, whereas human leukocyte antigen (HLA)‐related genes showed the highest expression in cluster C. (Figure 3D).

When summarizing the above, Cluster A was an “immune hot” group characterized by active inflammatory reaction and high immune checkpoint genes expression, whereas Cluster C was an “immune cold” group characterized by low immune activity and exceptionally high expression of *LAG3* and MHC class II‐related genes. The gene expression pattern of Cluster B was intermediate between Clusters A and C, however, showed characteristics closer to Cluster C.

### 3.4 Immunohistochemistry analysis for PD‐L1 and LAG‐3

**FIGURE 4:**
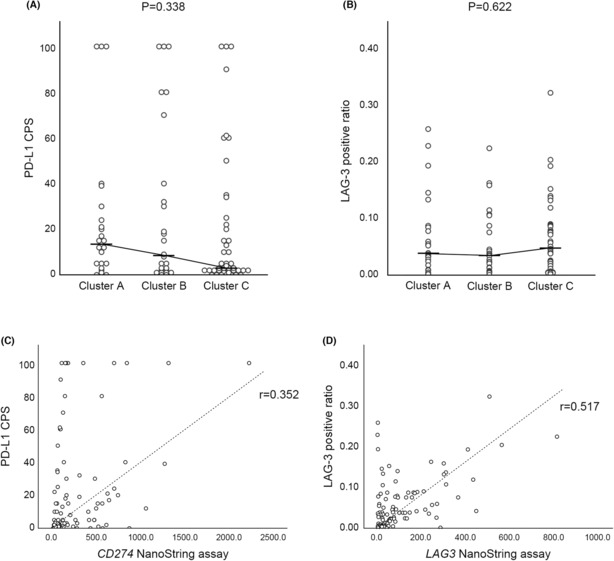
(A, B) Scatter plots showing the results of PD‐L1 (A) and LAG‐3 (B) immunohistochemistry for each cluster. Both markers showed no statistically significant differences according to clusters. (C, D) Scatter plots showing the correlation between immunohistochemistry and NanoString assay results in *CD274* (PD‐L1) (C) and *LAG3* (LAG‐3) (D). Both genes showed modest correlations between two methods. (CPS, combined positive score).

The average CPS of PD‐L1 was 22.41 (range, 0–100; standard deviation [SD], 31.736). The median values of PD‐L1 CPS were 13.50, 8.50, and 3.00 in clusters A, B, and C, respectively (*p* = 0.338). (Figure 4A) The average LAG‐3 positive ratio was 0.0622 (ratio, 0.0004–0.3189; SD 0.06408). The two cases in which LAG‐3 was diffusely expressed in the tumor cells were excluded from this analysis. The median values of the LAG‐3 positive ratios were 0.0381, 0.0343, and 0.0478 in clusters A, B, and C, respectively (*p* = 0.622). (Figure 4B) Scatter plots comparing PD‐L1 CPS with *CD274* expression values in the NanoString assay (Pearson's correlation = 0.352; *p* < 0.001), and LAG‐3 positive ratio with *LAG3* expression values in the NanoString assay (Pearson's correlation = 0.517; *p* < 0.001) are shown in Figure 4C,D, respectively.

## 4 DISCUSSION

In this study, we classified the TIME of DLBCL into three subgroups according to the results of the NanoString nCounter assay. Cluster A showed a relatively high activity of T‐cell‐associated immune reactions. The expression of almost all immune checkpoint genes, including *CD274*, increased in cluster A, but *LAG3* expression was the lowest in A. In contrast, cluster C showed the lowest immune activity among the three groups. While most of the immune checkpoint genes showed the lowest expression, *LAG3* showed the highest expression in cluster C, contrary to the expression patterns of almost all other immune checkpoint genes. Despite the limitation that the NanoString assay does not discriminate between tumor cells and non‐tumor immune cells, Clusters A and C showed relatively clear differences in gene expression patterns in the TIME of DLBCL.

LAG‐3 is mainly expressed in T‐cells and is also expressed in natural killer (NK) cells, and some subsets of plasma cells.^24^, ^25^ LAG‐3 shares significant homology with CD4 and binds to MHC class II molecules with approximately 100 times stronger affinity than CD4.^26^ This process can prevent CD4‐positive T‐cells from receiving antigen information from antigen presenting cells (APC), thereby inhibiting the host immune response against the antigen. When LAG‐3 is overexpressed in T‐cells around tumor cells (tumor infiltrating lymphocytes; TIL), it can contribute to immune evasion of tumors by inhibiting the recognition of tumor antigens by the host immune system.

In a study conducted on TIL of DLBCL, LAG‐3 showed higher expression in CD4‐positive regulatory T‐cells (Treg) and CD8‐positive T cells than in CD4‐positive non‐Tregs.^27^ Contrary to our observation that the expressions of PD‐L1 and LAG‐3 were negatively correlated, LAG‐3 was modestly correlated with PD‐L1 expression in this study. In contrast, in a paired longitudinal tumor tissue analysis performed on cHL, CD8‐positive T‐cell depletion and increased LAG‐3 expression were observed after anti‐PD‐1 therapy.^28^ In the cell surface analysis of CD4‐positive T‐cells, LAG‐3 expression was significantly higher in the patient samples treated with PD‐1 than in the control group. In our analysis, LAG‐3 showed the highest expression in cluster C, with the lowest cytotoxic T‐cell activity. Although it is assumed that the TIME of cHL and DLBCL is not identical, LAG‐3 can be suggested to be a uniquely activated immune checkpoint in the immune depleted microenvironment of lymphomas.

In our NanoString assay results, the activity of the *HLA* genes associated with MHC class I and class II molecules was relatively higher in cluster C than in clusters A and B, although the overall inflammatory activity was low. This observation suggests that when LAG‐3 is blocked with an anti‐LAG‐3 antibody in DLBCL with cluster C, CD4 can bind to abundant MHC class II molecules on APC without competition with LAG‐3 and receive sufficient information about the antigen. This might hinder the immune evasion by tumor cells by increasing the activity of tumor‐specific host immunity. The anti‐human LAG‐3 antibody relatlimab was first introduced in 2019.^29^ Although there are few clinical trial results for anti‐LAG‐3 antibodies in lymphoma, several studies have experimentally shown the potential of LAG‐3 as a target for DLBCL treatment.^30^, ^31^ In chronic lymphocytic leukemia patients, treatment of peripheral blood mononuclear cells with relatlimab depleted leukemia cells and restored NK‐cell and T‐cell‐mediated responses.^32^ The use of anti‐LAG‐3 inhibitors has been proposed as a combination therapy for synergistic effects with anti‐PD‐1 inhibitors.^33^, ^34^, ^35^ The use of combination therapy with the two antibodies has been suggested in various malignancies, such as breast cancer,^36^ glioblastoma,^37^ and renal cell carcinoma.^38^

In a clinical trial in patients with previously untreated metastatic or unresectable melanoma (RELATIVITY‐047), inhibition of two immune checkpoints, LAG‐3 and PD‐1, provided a greater benefit with regard to progression‐free survival than inhibition of PD‐1 alone.^39^ In March 2022, nivolumab (anti‐PD‐1 inhibitor) plus relatlimab received its first approval in the USA for the treatment of unresectable or metastatic melanoma in adult patients and pediatric patients aged ≥12 years who weigh ≥40 kg. Recently, a bispecific antibody capable of engaging LAG‐3 and PD‐L1 was developed, and clinical trials for this drug are underway.^40^ A treatment strategy that simultaneously blocks the LAG‐3 and PD‐1/PD‐L1 axis can also be applied to CAR‐T therapy. Studies using CRISPR‐Cas9 mediated gene editing demonstrated that the knockout of PD‐1 and LAG‐3 in CAR‐T cells overcomes the immunosuppressive nature of the TIME, a key factor limiting CAR‐T efficacy.^41^, ^42^, ^43^, ^44^

In our study, immunohistochemical staining of PD‐L1 and LAG‐3 did not correlate well with the expression levels of *CD274* and *LAG3* genes, respectively, and did not help classify clusters A, B, and C. Therefore, we suggest trying a treatment strategy using a combination of anti‐PD‐1 and anti‐LAG‐3 blockade for all DLBCL patients requiring immunotherapy, rather than using them individually for the patients in each TIME cluster.

In 2021, Kotlov et al. defined the TIME of DLBCL into four major categories, germinal center‐like, mesenchymal, inflammatory, and depleted, with distinct biological properties and clinical behavior through a transcriptomic analysis of 4655 DLBCLs.^45^ Although our results based on NanoString assay could not be directly matched with those of Kotlov et al., considering that some of the four groups presented in that study were classified by inflammatory activity of TIME, it is hoped that further studies can be made to compare our results with those of Kotlov et al.

In summary, DLBCL can have various TIME. In a subgroup with TIME with active host immune reaction, most immune checkpoints, including PD‐L1, are highly expressed. In contrast, in another subgroup with TIME with depleted host immunity, the activity of most immune checkpoints is low, while the expression of LAG‐3 is increased. Our results show that the unique expression pattern of LAG‐3 in DLBCL contrasts with that of other immune checkpoints. The unique expression pattern of LAG‐3 in DLBCL shown by our analysis suggests that the combination of anti‐PD‐1/PD‐L1 and anti‐LAG‐3 inhibitors in the immunotherapy of DLBCL patients can have a synergistic effect and broaden the range of the TIMEs covered by immunotherapy, improving the immunotherapy efficacy and outcome in DLBCL patients. In addition, not only the administration of immunotherapeutic agents, but also immune editing that allows CAR‐T cells to ignore the inhibitory signals from PD‐L1 or LAG‐3 can also open new avenues for DLBCL immunotherapy. We need further studies on applying the synergistic dual checkpoint blockade of PD‐1/PD‐L1 and LAG‐3 to treat DLBCL.

## AUTHOR CONTRIBUTIONS

**Hyunjee Lee:** Formal analysis (equal); writing – original draft (equal). **Sang Eun Yoon:** Resources (equal). **Seok Jin Kim:** Resources (equal). **Won Seog Kim:** Resources (equal). **Junhun Cho:** Conceptualization (lead); formal analysis (equal); supervision (lead); writing – original draft (equal); writing – review and editing (lead).

## CONFLICT OF INTEREST STATEMENT

All authors declare no competing interests.

## Data Availability

The datasets used and/or analysed during the current study are available from the corresponding author on reasonable request.
